# Supplementary material for: Are carbon emissions trading and green financial instruments synergistic? -Comprehensive quantitative research based on content analysis
Source: PLoS One. 2024 Mar 7;19(3):e0298601. doi: 10.1371/journal.pone.0298601 (PMC10919601; doi:10.1371/journal.pone.0298601)
Supplement: S1 Data — (ZIP) [file pone.0298601.s001.zip › Data/地级市Z_F工作报告.zip!/╕ú╜¿/╚2├≈╩╨/└┤╫╘╣1⁄2╓┌║┼í╛┬φ┐╦╩2╛▌═°í┐_2015.docx]

各位代表：

　　现在，我代表三明市人民政府向大会作政府工作报告，请予审议，并请市政协各位委员和其他列席人员提出意见。

　　一、2014年工作回顾

　　过去一年，我市各级政府在省委、省政府和市委的正确领导下，在市人大及其常委会、市政协的监督支持下，深入学习贯彻党的十八大、十八届三中、四中全会和习近平总书记系列重要讲话及来闽考察重要讲话精神，扎实抓好稳增长、调结构、促改革、惠民生等各项工作，经济社会发展取得新成效。初步统计，全市生产总值1621.21亿元，增长9.6%；规模以上工业增加值805.8亿元，增长12.2%；地方公共财政收入90.92亿元，增长1.2%；固定资产投资1603.08亿元，增长20.2%；外贸出口17.78亿美元，增长29.4%；实际利用外商直接投资1.4亿美元，增长12.3%；社会消费品零售总额404.63亿元，增长12.3%；城镇居民人均可支配收入25197元，增长10.1%；农村居民人均可支配收入11665元，增长10.8%；居民消费价格总水平上涨2%；节能减排年度任务可以完成。一些重要领域、重点项目实现突破：

　　——三明沙县机场基本建成，中心城市快速通道一期建成通车，三明城市绿道一期基本建成。

　　——入闱全国生态城市竞争力十强，获得省级生态市称号；被列为国家扶贫改革试验区。

　　——通用产业园、中科动力、福建月兔空调等一批重大项目和全省首个国家农业综合开发现代农业园区试点项目落地。

　　——春舞枝集团在德国证券交易所挂牌上市，成为全国首家花卉上市企业；南方制药成为我市首家在“新三板”挂牌上市企业。

　　——被确定为公立医院改革国家联系试点城市，国家公共文化服务体系示范区创建迈出实质性步伐，万寿岩遗址保护工程取得重大进展，义务教育标准化学校建设目标完成率和综治“三率”测评位居全省前列。

　　一年来的主要工作和成效是：

　　（一）集聚要素稳增长。面对经济下行压力，充分发挥政策引导作用，认真落实中央和省上稳增长促发展各项政策，制定出台稳定工业增长、支持企业技改、优化金融服务、促进房地产市场健康发展等政策，支持实体经济发展。启动“十百千”重大项目工程五年计划，实施年度重点项目2416个，完成投资1359亿元，788个项目建成或部分建成。加强“三维”项目对接，新签约项目215个，总投资701.8亿元。强化企业帮扶，开展“抓落实、解难题、促发展”活动，加强资金、用地、用工等保障，对困难企业一对一帮扶，助推企业加快发展。积极开展融资协调，建立市县两级领导挂包重点风险企业制度，有效防范和化解金融风险。

　　（二）调整结构壮产业。实施产业龙头促进、产业链拓展延伸计划，全市培育形成2家产值超百亿、23家产值超十亿元企业，6条产业链产值突破2000亿元。企业技改力度加大，完成投资占全省技改投资近两成。中机院海西分院、中节能环保产业园等项目加快建设，稀土、氟化工、新能源汽车等新兴产业加快发展。出台加强矿产资源勘查开发管理意见，推动矿产资源向龙头企业集中、往产业发展提升。实施现代农业发展行动计划，新增家庭农场、合作社1243家，新增土地流转面积3.6万亩，市级以上农业龙头企业完成产值330亿元。实施120个服务业重点项目，文化产业主要指标增幅居全省前列，三明郊野地质公园和清流温泉地质公园获评国家地质公园，新增4家4A级旅游景区。推动电子商务加快发展，淘宝网特色中国三明馆开馆，全省首个阿里巴巴农村淘宝项目落户尤溪，涌现出一批年销售额超亿元的电商企业。

　　（三）统筹城乡促宜居。实施城乡基础设施六项提升工程，厦沙高速公路全线开工，吉泉铁路前期工作取得突破，205国道、306省道改线工程加快推进，市区第二供水工程动工建设。出台进一步放宽户口准入条件、推进农业转移人口市民化等政策措施，公共服务稳步向常住人口覆盖。加快生态工贸区生态新城建设，联办园区建设力度加大，市区与沙县、永安在基础设施、产业、规划等方面同城化迈出新步伐。实施宜居环境建设行动计划，统筹推进县城、小城镇和美丽乡村建设，泰宁县荣获全省首批国家生态文明建设示范区称号，5个县（市）荣获省级生态县（市）称号，尤溪洋中镇列入全省“小城市”培育试点。环境保护扎实推进，流域整治成效明显，造林绿化、水土流失治理超额完成年度任务。全境列入赣闽粤原中央苏区振兴发展规划，苏区老区加快发展。

　　（四）改革开放增活力。创新林业金融产品和风险防控机制，在全国首推林权按揭贷款，成立全省首家林权收储中心。交通银行三明分行开业，新增41家企业在海峡股权交易中心挂牌，4家基金公司落地运作，永安成功发行城投债10亿元，便民基础金融服务和阳光信贷标准化网点实现行政村全覆盖。福建一建集团有限公司挂牌成立。市县政府机构改革有序推进。启动实施工商登记制度改革。优化外贸发展环境，外贸出口保持较快增长。支持民营经济发展，出台推进明商回归工程意见，民间投资占全市固定资产投资的81.5%。深化明港澳台侨合作，成功举办第十届林博会和第七届海峡两岸客家高峰论坛。

　　（五）改善民生保稳定。21项为民办实事项目全面完成，全市民生支出占公共财政支出的比重达78.2%。全市新增城镇就业2.87万人，农村劳动力转移就业4.74万人，城镇登记失业率2.1%。城乡居民养老保险基础养老金、城乡低保标准、退休劳模待遇进一步提高。保障性安居工程新开工5753套，基本建成7793套，超额完成年度任务。完成造福工程搬迁和危房改造8850户、3.88万人。强化科技和人才工作，出台6个人才政策性文件，2人入选国家创新人才推进计划，新组建3家院士工作站，市科技馆建成开放。农村中小学生“幸福成长工程”持续深化，5个县（市、区）被认定为义务教育发展基本均衡县。全国城市公立医院综合改革试点座谈会在我市召开，医疗卫生服务体系逐步完善，群众看病负担进一步减轻。市“两馆一宫”建成，4个镇（村）列入第六批中国历史文化名镇（村）。“单独两孩”政策顺利实施，实行新生儿落户与计生脱钩，全面完成人口计生年度责任目标。群众体育广泛开展，我市运动员在世锦赛、亚运会等大赛中获得2金3银1铜。全力争创全国文明城市，纪念全国“五讲四美三热爱”活动30周年研讨会在我市举行。深化“平安三明”建设，抓好信访工作，加强安全生产，保障食品药品安全。老龄、工会、青少年、妇女儿童、残疾人工作持续推进，司法行政、统计、科普、民族宗教、档案、地方志、水文、气象、防震、库区移民工作取得新进展。人民防空、民兵预备役、国防后备力量建设和双拥工作进一步加强。

　　（六）转变作风提效能。深入开展党的群众路线教育实践活动，认真贯彻落实中央八项规定精神，文风会风得到改进，“三公”经费支出下降35.9%。取消和下放市级行政审批114项，市级行政审批事项保留数量全省最少。整合升级网上办事大厅，推行网上直接审批，全省首个市级中介服务中心投入运行，全市142个乡镇（街道）全部建成便民服务中心。大力推行“马上就办”，机关效能建设和绩效管理得到加强。坚持依法行政，自觉接受各方监督，广泛听取各方意见，办好人大代表建议和政协提案，推进政务公开和政府信息公开，强化行政监察和审计监督，勤政廉政建设取得新成效。

　　各位代表，过去一年的成绩来之不易，是在省委、省政府和市委正确领导下，经过全市上下奋力拼搏取得的。在此，我代表市人民政府，向全市人民，向人大代表、政协委员、各民主党派、工商联、各人民团体、无党派人士、离退休老同志和社会各界人士，向中央和省属驻明单位、驻明部队、武警官兵、政法干警，向关心支持三明发展的港澳台同胞、海外侨胞、国际友人，致以衷心的感谢和崇高的敬意！

　　在看到成绩的同时，我们也清醒地认识到，我市经济社会发展中还存在不少困难和问题，主要是：保持经济持续较快发展的基础还不牢固，龙头企业偏少，大项目、好项目不多，部分企业生产经营困难，财政收入增幅放缓，房地产、金融信贷风险增大。工业经济规模偏小、结构不优，三产业态不多、档次偏低，农业产业化程度不高，科技创新能力较弱，生态保护和环境治理压力加大。城镇化水平偏低，市区经济发展不快，农村公共服务和基础设施薄弱。社会保障水平与群众期待还有差距，安全生产、食品药品安全、社会治理还需加强。政府工作有待进一步改进，一些部门执行力、落实力不强，“庸懒散拖”现象依然存在。对此，我们要高度重视，采取有力措施加以解决。

　　二、2015年主要任务

　　今年是全面深化改革的关键之年，是全面推进依法治市的开局之年，也是全面完成“十二五”规划的收官之年。政府工作的总体要求是：全面贯彻党的十八大、十八届三中、四中全会和中央经济工作会议精神，深入学习贯彻习近平总书记系列重要讲话和来闽考察重要讲话精神，按照省委、省政府和市委部署，主动适应经济发展新常态，坚持稳中求进工作总基调，坚持以提高经济发展质量和效益为中心，坚持“念好发展经、画好山水画”工作主题，深化改革开放，突出创新驱动，做大产业龙头，加强民生保障，推进依法治市，进一步加快三明科学发展跨越发展，为建设机制活、产业优、百姓富、生态美的新福建作出新贡献。

　　今年我市经济社会发展主要预期目标是：全市生产总值增长10.5%左右；地方公共财政收入增长10%左右；固定资产投资增长18.5%左右；外贸出口增长7%，实际利用外商直接投资增长7%；城镇登记失业率控制在3.8%以内；社会消费品零售总额增长14%，居民消费价格总水平涨幅控制在3%左右；人口自然增长率控制在9.5‰以内；城镇居民人均可支配收入增长9.5%，农村居民人均可支配收入增长10%；完成单位生产总值能耗降低和主要污染物减排年度任务。围绕上述目标，突出做好七个方面工作：

　　（一）着力投资拉动，持续促进稳定增长

　　加强基础设施建设。交通方面，全力推进南龙铁路建设，启动三明南站站房建设，确保吉泉铁路、力争浦梅铁路动工，抓紧鹰厦线台江至荆西段改线工程；建成莆炎高速三明莘口至明溪城关段、漳永高速三明段，加快厦沙高速公路建设，力争开工莆炎高速尤溪中仙段，续建和新建国省干线及镇镇有干线公路252公里；确保三明沙县机场实现通航。能源方面，加快建设海西天然气管网三明段一期工程，开工建设华电宁化风能发电、泰宁池潭水电厂扩建等项目，推进三明核电、沙县热电联产等项目前期工作；建成2项220千伏输变电工程，继续实施农网改造升级工程。市政方面，实施“五千工程”，推进城市地下综合管廊建设，完成市区第二供水工程，加快列东大桥改造等项目。水利方面，推进明溪黄沙坑、尤溪兴头、沙县双溪、永安溪源等水库建设，实施一批重点流域防洪工程。

　　深化“三维”项目对接。央企项目重点加快中机院海西高端装备产业园、中节能环保产业园、通用产业园等项目建设，推动中石化含氟润滑油等项目尽快落地，力争完成投资13.5亿元以上。民企项目重点盯紧全国民企500强和行业龙头，力争新签约合同项目投资额300亿元以上，新签约项目动工率50%以上。外企项目重点对接台湾百大、大型港澳侨企业，抓好省上跟踪管理的11个重点项目，力争新签约合同项目利用外资3.5亿美元以上。

　　全力保障重点项目建设。突出抓好464个列入省行动计划的重大项目，实施“十百千”项目2458个，完成投资1250亿元以上，建成或部分建成600个以上。加强重大项目策划，做深做实前期工作，建立健全重大项目库，完善协调推进、跟踪服务和督查考评机制，狠抓审批、开工、竣工各个环节，着力解决征迁、资金等难题，推动项目早开工、早建成。全面推行标准化管理，打造阳光工程、优质工程。高起点、高标准编制“十三五”规划。

　　（二）强化产业支撑，持续壮大实体经济

　　促进工业提质增效。落实工业发展扶持政策，加大对企业要素保障、市场拓展等帮扶力度，优化金融服务、强化融资协调，鼓励企业重组改造，全力稳住工业基本面。实施产业龙头促进计划，重点扶持三钢、海西重汽、金明稀土、三农氟化工、中科动力等50家龙头企业，力争产值突破700亿元。拓展延伸6条产业链，实施150个产业链重点项目，推动20万吨离心球墨铸铁管、4000吨钴酸锂等一批项目建成投产。实施300个以上投资超千万元重点技改项目，完成投资170亿元以上，建成投产或部分投产200个以上。加快发展新材料、新能源、节能环保、生物医药、高端装备制造等战略性新兴产业。推动信息化与工业化深度融合，实施一批示范项目。加强工业园区建设，完善园区基础设施，积极推进“二次招商”。

　　加快第三产业发展。大力发展商贸流通业，合理规划建设城市商贸综合体，改造提升传统流通业态，完善仓储、冷链等物流设施，建设区域性物流节点城市。加快旅游业发展，支持泰宁开展全省旅游综合改革试点，推动市域主要景区联动发展，加强重点旅游项目、旅游线路建设，创新旅游市场营销，鼓励发展民宿旅游，打造市区旅游集散服务中心。实施一批重点文化产业项目，发展文化旅游、文化创意、工艺美术等产业，提升文化产业实力。加快电子商务发展，着力培育各类电商主体，支持清流花木交易所、海鑫钢材信息网、林品汇、淘宝网特色中国三明馆等平台建设，完善电商配套体系，促进线上线下融合发展，创建国家电子商务示范城市。围绕培育新的消费增长点，大力发展医疗保健、家政、会计、信息、咨询、会展等服务业，做大总部经济、楼宇经济。落实促进房地产业发展政策，引导房地产市场平稳健康发展，满足多样化住房需求。落实扶持建筑业发展壮大措施，提升建筑业企业竞争力。

　　坚持科技创新驱动。强化科研市场导向，落实鼓励创新政策，突出企业创新主体地位，深化产学研用合作，促进科技成果转化和产业化。实施70个以上重大科技项目，推进中机院、中节能、中纺院科技孵化器建设，新增省级工程技术研究中心、技术公共服务平台及科技企业孵化器10家以上，新组建院士工作站3家以上。加快高新技术产业发展，推进三明高新技术产业开发区升格为国家级高新区。支持企业制修订行业标准和创建品牌，加强知识产权保护。加大人才政策落实力度，大力培养本土人才，推进6条特色产业链人才集聚，吸引科技创新团队和人才到我市创业发展。

　　（三）夯实“三农”基础，持续增加农民收入

　　大力发展特色现代农业。保障粮食安全，全面落实惠粮政策，从严保护耕地，确保粮食播种面积稳定在320万亩、总产稳定在110万吨以上。做大做强高优粮食、绿色林业、精致园艺、生态养殖、现代烟草等五大特色优势产业，扶持发展设施农业，发展林下经济和休闲观光农业。加快“一区三园”建设，落实用电、用地等扶持政策，推动农产品加工企业向园区集聚，提升农业产业化水平。加强农业科技创新和推广，提高农作物耕种收综合机械化水平，推进建宁国家级制种基地建设。健全农业保险服务体系，抓好重大动植物疫病防控工作，完善农产品质量安全可追溯管理平台，加快农业标准化生产，保障农产品质量安全。

　　构建新型农业经营体系。推进农业经营组织再造，规范提升农民合作社，扶持发展家庭农场，壮大农业龙头企业，培育一批新型职业农民。完善各类新型农业经营主体与农户的利益联结机制。抓好农村土地承包经营权确权登记颁证工作，稳定农村土地承包关系。放活土地经营权，发展股份合作、土地托管、信托流转等规模经营模式，完善市县乡村四级土地流转服务平台，健全公开规范的土地流转市场。探索建立农村产权综合交易中心，激活农村生产要素。

　　实施科学扶贫和精准扶贫。用好苏区老区政策，推进三明国家扶贫改革试验区建设。推广"348"精准扶贫工作机制，突出抓好5个省级扶贫开发工作重点县，推进100个扶贫开发重点村建设，落实一批精准扶贫重点项目，力争减少贫困人口2万人以上。实施造福工程搬迁和危房改造4000户以上，解决4.7万农村人口饮水安全问题。加快少数民族乡村发展，加强库区移民安置和后期扶持。

　　（四）推进城乡联动，持续打造宜居环境

　　加强城市建设。推进市区与沙县、永安同城化，健全完善生态工贸区管理体制机制，突出抓好9个重点园区开发，加快生态新城“一带六区”建设，推动生态新城创建国家低碳城。抓紧中心城市快速通道二期工程和205国道、306省道改线工程建设。抓好小蕉工业园扩园和黄砂化工园、新型建筑材料产业园、铁路装备产业园等建设，大力发展城市型产业，进一步向两区放权，推进贵溪洋、徐碧、台江、富兴堡等重点板块开发，实施“沙溪十里闽学文化长廊”、城市绿道二期等项目。深入推进“大城关”战略，引导各县因地制宜加快新型城镇化，注重文化传承和特色发展，强化产城联动，推动县城扩容提质、做强做美。

　　创新体制机制。强化规划引领，推行“多规合一”。有序推进农业转移人口市民化，大力推行无门槛居住证制度，把进城落户农民纳入城镇住房和社会保障体系，推进基本公共服务向常住人口覆盖。深化小城镇综合改革，稳妥推进强镇扩权，用好城乡建设用地增减挂钩、土地整治、三旧改造、财税和投融资等配套政策，加快4个省级试点镇和16个市级中心乡镇改革发展，支持尤溪洋中镇开展“小城市”培育试点，提升小城镇发展水平。

　　推进城乡环境建设。延伸拓展“点线面”攻坚，提升城镇“三边三节点”整治水平，开展农村生活垃圾专项治理。抓好快速铁路、高速公路沿线环境整治，推广城市立体绿化和片林建设，整治村庄130个以上，推进100个美丽乡村示范村建设，打造20个城市完整社区。加强历史文化名镇名村、历史建筑、传统村落保护。强化城镇综合管理，完善“数字城管”，深化“两违”综合治理，健全整治环境卫生、交通秩序等长效机制，促进城镇管理常态化、规范化、精细化。

　　（五）深化改革开放，持续激发内生动力

　　抓好重点领域改革。深化投资体制改革，进一步削减投资项目前置审批事项，推进工商登记制度改革，落实支持民营经济发展政策措施，积极发展混合所有制经济。完善集体林权制度改革，推进联户证分发到户工作，培育新型林业经营主体，扩大林权抵押贷款规模，规范林权流转，开展重点区位商品林补偿赎买试点。深化金融改革，推广沙县农村金融改革经验，抓好村级融资担保基金建设，争创全国农村金融改革创新试验区。实施“引银入明”工程，力争新引进4家区外银行，鼓励投资基金业发展，实现村镇银行县域全覆盖。支持企业上市融资，力争1-2家企业实现上市，推动有条件的企业到“新三板”、海峡股权交易中心等市场挂牌，鼓励企业通过发行债券、资产证券化、引入风险投资等方式融资，积极推行PPP模式。加强金融生态建设，依法打击非法集资和恶意逃废债，着力防控金融风险。深化预算管理制度改革，建立跨年度预算平衡机制，全面实行全口径预算。稳步推进政府机构改革，有序推进事业单位分类改革。积极推行政府购买服务。完成机关公务用车制度改革。

　　扩大对外开放。积极融入福建自贸区，主动承接自贸区“外溢”效应。用好扶持外贸发展政策，促进外贸转型升级，推动生产型企业自营出口，扩大地产品出口。加快三明陆地港二期工程和保税仓库建设，提高通关便利化水平。加强对外经贸合作，支持企业设立境外营销网点，拓宽利用外资领域，鼓励外资企业增资扩股。深化明台交流，办好第十一届林博会，加强明台乡镇和产业合作，推动明台经济、文化、教育、生活深度融合。拓展区域协作，推动建立更加紧密的合作机制。密切与港澳地区、国外友城的沟通联系，做好与海外华侨华人及其社团的联络工作。

　　（六）加强环境保护，持续建设生态文明

　　强化生态建设。推进三明南方林区生态文明建设综合配套改革，做好生态功能红线划定工作，建立健全生态文明制度体系。加快国家级生态市创建工作，力争6个县（市）通过国家级生态县（市）技术评估，建成国家环保模范城市，基本达到国家森林城市创建标准。加强闽江源生态保护，强化自然保护区、重要湿地、饮用水源地等重点生态功能区保护，抓好矿山生态保护与修复，持续推进“四绿”工程，完成造林绿化29.8万亩，治理水土流失24.5万亩。节约集约利用土地，鼓励开发利用地下空间。

　　强化源头防控。严格环境影响评价，严控高能耗、高排放项目。实施50项节能技术改造和循环经济项目，大力推行清洁生产，加大落后产能淘汰力度。实行污染物排放总量控制，加快重点减排工程建设，推进工业园区污染集中处理和集中供热，抓好重点行业脱硫脱硝工作。实施市区、县城污水处理厂扩能工程，全面建成重点流域1公里范围内污水处理设施。推进生态产品市场化建设，抓好排污权有偿使用和交易工作，探索开展碳排放权交易。

　　强化环境整治。突出大气污染防治，抓好机动车尾气、施工扬尘、油烟、工业废气综合治理，支持三钢、三化等企业加大污染治理力度，提高市区空气质量。全面落实“河长制”，深入开展闽江流域水环境综合治理。强化畜禽养殖污染整治，推进科学养殖、生态养殖。实施土壤污染治理与修复工程。严厉打击各类环境违法行为，提高防范环境风险能力。

　　（七）坚持民生优先，持续维护和谐稳定

　　完善社会保障制度。落实就业创业各项扶持政策，统筹做好大中专毕业生、农村转移劳动力、城镇困难人员、退役军人就业工作，城镇新增就业2.8万人以上、农村劳动力转移就业4.5万人以上。加大社会保险扩面力度，加强社会保障基层基础建设，稳妥实施机关事业单位养老保险制度改革。提高企业退休职工养老保险待遇和城乡居民基础养老金，提升优抚对象抚恤和生活补助、城乡低保补助水平。新开工保障性安居工程4800套，基本建成3800套，规范分配和运营管理。创新养老模式，发展养老服务产业。完善未成年人、农村留守儿童、妇女、残疾人关爱服务体系，大力发展社会福利、社会救助、红十字会、慈善、老龄事业，支持关心下一代工作。

　　优先发展教育。坚持立德树人，把培育和践行社会主义核心价值观融入国民教育全过程，加快市中小学生示范性综合实践基地建设，全面推进素质教育。实施第二期学前教育三年行动计划，新建和改扩建一批公办幼儿园，加强民办幼儿园管理。实施义务教育标准化学校建设水平提升工程和城区中小学扩容改造工程，全面完成义务教育发展基本均衡县创建目标。加快普通高中特色多样化发展。大力发展现代职业教育，加强紧缺技能型人才培养，加快市职教园区建设。推动高校内涵发展，支持三明学院建设高水平应用技术大学、三明职业技术学院创建省示范性高职院校。加强教师队伍建设。发展特殊教育、民族教育、社区教育、老年教育。重视科普工作，增强全民科学素质。

　　提升全民健康水平。深化医药卫生体制改革，继续完善市县公立医院综合改革，推进基层医疗卫生机构第二轮改革，优化医疗资源布局，进一步理顺医疗服务价格，落实分级诊疗制度，建立多元化投入机制，积极引导社会资本办医。加强公共卫生体系建设，提升疾病防控和医疗卫生服务保障水平。坚持计划生育基本国策，提高出生人口素质，推进诚信计生·幸福家庭全国试点市创建，深化生育关怀。扎实开展全民健身运动，提升竞技体育实力，发展体育产业，充分发挥老体协作用，加快市少体校新校区和全国首个大体操培训基地建设。

　　促进文化繁荣发展。推进国家公共文化服务体系示范区创建工作，继续实施文化惠民工程，推行“六联六创”服务方式，扎实抓好公共文化服务标准化、基层综合性文化服务中心建设、公共文化机构法人治理结构等试点工作。鼓励社会力量参与提供公共文化服务、举办重大文化活动。推进市级广电管理运营机构改革，加快组建三明广播电视台。强化文化体育场馆公益性使用，积极申办各类国家、省级重大赛事活动。重视文化遗产的保护和传承，加快万寿岩国家考古遗址公园建设。深入挖掘特色文化，鼓励创作文化精品。繁荣社会科学，发展文学艺术。抓好第二轮修志工作。

　　加强社会治理创新。深化精神文明创建，巩固提升创城成果，认真做好迎接全国城市文明程度指数和全国未成年人思想道德建设年度测评工作。推进“平安三明”建设，创新立体化社会治安防控体系，加强城乡社区网格化管理和流动人口服务。完善信访工作机制，抓好矛盾纠纷排查调处，积极化解信访积案。深化安全生产标准化建设，强化道路交通安全综合整治，持续开展“打非治违”专项行动，坚决遏制重特大安全事故。严格食品药品安全监管，保障群众饮食用药安全。注重突发公共事件应急能力建设，继续抓好防灾减灾工作。健全完善社会信用体系。推进基层民主自治，做好村（居）委会换届选举工作。密切与各民主党派、工商联、无党派代表人士的联系与协商，支持工会、共青团、妇联等人民团体发挥桥梁纽带作用。发展外事侨务、民族宗教事业。深化双拥共建，加强国防动员、人民防空、民兵预备役工作，推进军民融合发展。

　　三、深入推进依法行政，加快建设法治政府

　　党的十八届四中全会明确提出了建设法治政府的要求，我市各级政府各部门要在党的领导下、在法治轨道上开展工作，把依法行政要求贯穿到政府工作全过程、各领域，加快建设职能科学、权责法定、执法严明、公开公正、廉洁高效、守法诚信的法治政府。

　　更加注重依法。自觉接受人大及其常委会的法律和工作监督，自觉接受政协的民主监督，认真办理人大代表建议和政协提案。深入开展法治宣传教育，增强领导干部办事依法、解决问题用法、化解矛盾靠法的观念和能力。落实重大行政决策法定程序，建立行政机关内部重大决策合法性审查机制，推行政府法律顾问制度。严格依法制定规范性文件，体现权利公平、机会公平、规则公平。深化行政执法体制改革，合理配置执法力量，积极推行综合执法，加大关系群众切身利益的重点领域执法力度。加强行政复议和行政应诉工作，促进行政与司法良性互动，完善行政争议协调化解机制。

　　更加注重高效。巩固和拓展教育实践活动成果，持续深化作风建设，决不让“四风”问题反弹回潮。加大简政放权力度，全面清理非行政许可审批，推行行政权力清单制度，深化审批服务标准化建设，规范和优化中介服务，进一步激活市场潜力、增强创业动力。深入开展“马上就办”，加强行政服务中心建设，推行“一口受理”、“限时办理”，急事急办、特事特办。完善网上办事大厅，大力推进网上审批，多让数据网上走，少让群众路上跑。加快建设绩效管理信息系统，推进绩效管理规范化。深入清理各类达标评比表彰活动，严格规范涉企收费行为，为基层和企业进一步放权减负。

　　更加注重落实。紧盯发展形势变化，及时跟进应对举措，做到抢抓机遇不迟钝、解决问题不拖延、化解矛盾不积压。发扬“钉钉子”精神，对看准的事情、部署的工作、明确的任务，雷厉风行、一抓到底，确保各项工作落到实处、取得实效。深入推进“四下基层”，倡导践行“一线工作法”，多到困难大、矛盾多、条件差的地方，把问题解决在一线、工作完成在一线。鼓励支持领导干部敢于担当、敢于作为、敢于创新，坚决纠正不作为、乱作为，坚决克服懒政、怠政，坚决惩处失职、渎职，营造风清气正的干事创业氛围。

　　更加注重廉洁。厉行勤俭节约，反对铺张浪费，严控“三公”经费，进一步降低行政成本。深化政务公开，推进财政预算、公共资源配置、重大建设项目实施、社会公益事业建设等重点领域的信息公开。强化权力运行制约监督，加大对工程建设、矿产开发、土地出让和国有资产管理等领域的监管力度，推进电子化交易平台建设，防止权力乱用滥用。严格落实中央八项规定精神和廉洁从政各项规定，认真落实党风廉政建设责任制，加大行政监察和审计监督力度，严肃查处各类违纪违法案件，努力实现干部清正、政府清廉。

　　各位代表，做好今年工作，任务艰巨而光荣。让我们紧密团结在以习近平同志为总书记的党中央周围，在市委的坚强领导下，提振精气神、弘扬好作风、凝聚正能量，开拓进取、埋头苦干、攻坚克难，为全面完成今年目标任务、加快推进三明科学发展跨越发展而努力奋斗！
